# Asia’s Growing Contribution to Obesity Surgery Research: A 40-year Bibliometric Analysis

**DOI:** 10.1007/s11695-024-07138-z

**Published:** 2024-03-07

**Authors:** Ziyun Liu, Haiqin Wang, Dazhi Fan, Tingting Xu, Fuzhen Wan, Qing Xia

**Affiliations:** 1 International Business School Suzhou, Xi’an Jiaotong-Liverpool University, Suzhou, Jiangsu People’s Republic of China; 2 The International Peace Maternity and Child Health Hospital, School of Medicine, Shanghai Jiao Tong University, Shanghai, China; 3 Foshan Fetal Medicine Research Institute, Affiliated Foshan Women and Children Hospital, Southern Medical University, Foshan, 528000 Guangdong China; 4 Department of Obstetrics, Affiliated Foshan Women and Children Hospital, Southern Medical University, Foshan, 528000 Guangdong China; 5 Department of Health Management and Policy, School of Public Health, Capital Medical University, Beijing, 100069 China; 6 School of Public Health, Peking University, Beijing, 100083 China; 7 Department of Gastrointestinal Surgery, The Second Affiliated Hospital of Anhui Medical University, Hefei, Anhui People’s Republic of China; 8 Australian Centre for Health Services Innovation and Centre for Healthcare Transformation, School of Public Health & Social Work, Faculty of Health, Queensland University of Technology, Brisbane, QLD Australia

**Keywords:** Asia, Obesity surgery, Bariatric surgery, Bibliometrics, Sleeve gastrectomy

## Abstract

Bariatric metabolic surgery’s global research interest is growing, particularly in Asia due to its high obesity rates. This study focuses on Asia, especially China, analyzing 3904 publications (1221 from China) from 1980 to 2022. Research output accelerated until the COVID-19 pandemic, driven by economic growth and rising obesity rates. China led contributions from 2010, but Western Asia led when adjusted for population. An intra-regional research collaboration network emerged, driven by geographic proximity and similar economic environments. Keyword analysis highlighted emerging topics like “laparoscopic sleeve gastrectomy” and “non-alcoholic fatty liver disease,” indicating a shift in focus. The study recommends disseminating research in top-tier journals to enhance visibility and impact.

**Graphical Abstract:**

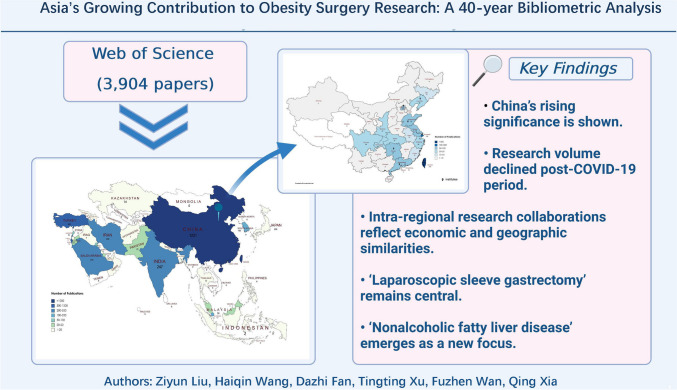

**Supplementary Information:**

The online version contains supplementary material available at 10.1007/s11695-024-07138-z.

## Introduction

The escalating global prevalence of obesity constitutes a severe public health crisis [1], with estimates indicating that over 1.5 billion adults could be obese by 2035 [2]. The obesity pandemic brings significant health ramifications, including an heightened risk of diabetes, cardiovascular diseases, and metabolic disorders [1]. Asian countries, such as China, Thailand, Korea, and Singapore, are increasingly grappling with this challenge, underscored by the rising prevalence of obesity [3]. China, for instance, records an adult obesity rate of 16.4%, which correlates with the increased incidence of premature mortality [4]. This situation necessitates immediate and effective interventions to mitigate the escalating problem.

Obesity surgery, also known as bariatric metabolic surgery (BMS), introduced by Kremen in 1954, has emerged as a potent tool in the fight against obesity [5]. It is renowned for its efficacy in facilitating sustainable weight loss and managing obesity-related comorbidities [6]. Owing to technological advancements and heightened demand, bariatric surgical techniques have continuously evolved across the globe [7]. Despite being relatively new in Asia, BMS is making significant contributions to the refinement of these procedures [8]. However, the current research landscape concerning BMS in Asia remains unclear, and there is a need for comprehensive and systematic scrutiny of research trends and focal points in these regions.

Bibliometrics is an interdisciplinary method that employs mathematical and statistical tools to quantitatively examine publications [9]. It enables the evaluation of literature performance within a specific domain and identification of influential authors, articles, primary journals, and publication trends. Additionally, it provides insights into scientific networks, collaborations, and connections through co-authorship, citation, co-citation, and co-occurrence analysis. Thus, bibliometric analysis can shed vital light on the BMS research landscape.

While previous studies have deployed bibliometric analyses to delineate research characteristics in BMS such as scientific publications, current research status, and future prospects [10–13], no such study has holistically explored this surgical field within the Asian context. Hence, this study seeks to bridge this gap by offering a comprehensive bibliometric analysis of BMS research in Asia, drawing upon scholarly publications dating back to 1954.

## Material and Methods

### Data Source

For this bibliometric study, we exclusively utilized the Web of Science (WoS) as the data source. Renowned for its extensive coverage, WoS indexes over 70 million items, upholding its rigorous indexing standards that enhance the reliability of citation analysis [14]. This ensures a degree of precision and relevance in data collection that is paramount for a study of this nature. While acknowledging the extensive scope of databases such as Scopus and PubMed, the focused objectives of our research necessitated a data source characterized by both authoritative content and consistent indexing practices [14]. WoS fulfills these requirements, offering a well-curated repository of significant publications that are central to evaluating Asia’s, growing influence in the realm of obesity surgery research [15].

### Search Strategy and Study Selection

The study utilized a strategic search methodology employing key terms such as “bariatric surgery,” “obesity surgery,” “sleeve gastrectomy,” “gastric band,” and “roux-en-y gastric bypass.” These keywords, along with their corresponding synonyms, were combined using the Boolean operator “OR.” A comprehensive list of the search strategy is available in Supplementary Table 1.

In terms of geographic delineation, articles were classified as Asian if the corresponding author had an affiliation with any of the 48 countries in Asia (https://www.worldometers.info/geography/how-many-countries-in-asia/). In the context of China, including Taiwan, Hong Kong, and Macao, publications were assigned to each province or municipal city according to the affiliation of the corresponding author. Publications were restricted to original research articles and review articles and only those published in English language were considered. According to the first recorded BMS took place in 1954 [5], the study’s temporal boundaries were set from 1954 to 2022, to account for incomplete indexing of 2023 publications in the WoS. The initial data extraction process was completed on 20 January 2023, with a follow-up update conducted on 11 April 2023.

### Bibliometric Analysis and Statistical Analysis

The selected publications were exported in a plain text format to facilitate further data analysis. The distribution of the publications across Asia was visually represented using MapChart (https://www.mapchart.net/index.html). For the bibliometric component, biblioshiny, a user-friendly application for the R-based bibliometrix package, was employed [16]. Pearson’s correlation was used to examine the relationship between the volume of publications and the economic prosperity as well as the population scale. A bubble heat map [17] was constructed to provide a visual representation of the most prolific authors and prevalent research topics. To analyze the contributions of top authors, a four-dimensional bubble heat map was created where the x-axis represented the chronological timeline, and the y-axis denoted the authors. The size and color of the bubbles indicated the number of articles published each year and the number of citations received within that year, respectively. Additionally, the top prolific journals, the most active institutions, and the most cited articles were also researched. To illustrate the interrelations within the data, VOSviewer (Version 1.6.19) was utilized to create bibliometric network visualizations [18]. Each node in the VOSviewer network represented a unique item, with its size reflecting frequency, and the lines between nodes indicating connections between items. Different clusters were depicted in different colors. In terms of the overlay map, recent items were shown in yellow, while older items appeared darker.

### Subgroup Analysis

China’s significant role in the field of BMS research has necessitated a targeted subgroup analysis to fully understand its contributions and its shaping effect on the research landscape across Asia. This focused approach allows us to identify key trends, leading research institutions, and influential authors in China, offering deep insights into the country’s pivotal role in advancing BMS. By examining China’s extensive literature on BMS independently, we uncover the nuances of its research dynamics, how it shapes regional scientific discourse, and its global impact on obesity surgery research.

## Results

### The Volume and Trend of Publications

**Fig. 1:**
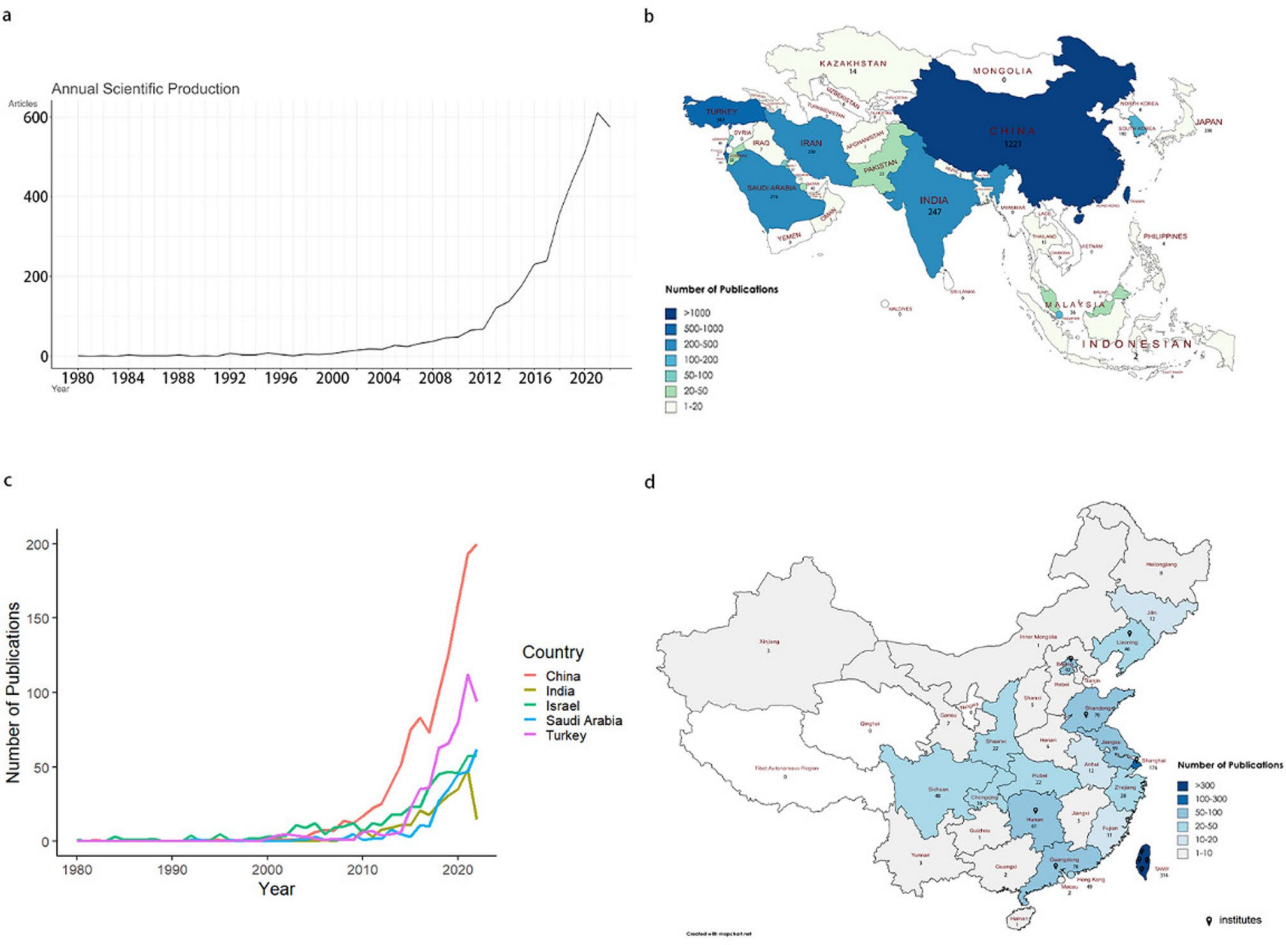
Bariatric metabolic surgery research publication trends in Asian countries. **a** Yearly publication count from 1980 to 2022. **b** Geographical distribution of publications across Asia. **c** Annual publication count of the top five prolific countries from 1980 to 2022. **d** Geographical distribution of publications across China

In total, *n* = 4669 articles were retrieved from WoS database. After manually deleting non-Asian publications (*n* = 731) or those published in 2023 (*n* = 34), a total of 3904 Asian BMS-related publications were identified between 1980 and 2022. Notably, these 43 years witnessed a significant upward trend in publication output on BMS, from a single paper in 1980 to 574 papers in 2022 (Fig. 1a). The publication frequency markedly surged around 2010, followed by a decline after 2020 (Fig. 1a).

Across 29 Asian countries contributing to the field of BMS research (*n* = 3904 in total), China emerged as the leading contributor with 1221 articles (Fig. 1b). China’s publications rose from a single paper in 1982 to 195 papers in 2022 (Fig. 1c). In fact, China’s annual publication volume displayed an upward trend, and consistently lead Asia in this respect since around 2010 (Fig. 1c).

### Top Prolific Countries or Provinces

Table 1 outlines the top 15 most prolific countries in Asia in terms of BMS research, and the full list of 29 contributing countries is shown in Supplementary Table 2. The bulk of the research emerges from East Asia and the Middle East. China (1221, 31.28% of 3904) leads the pack, followed by Turkey (562, 14.40%), Israel (502, 12.86%), Saudi Arabia (276, 7.07%), and India (247, 6.33%). Notably, China also topped the list in terms of citation frequency with a total of 18,009 citations, succeeded by Israel, India, Turkey, South Korea, and Japan.

**Table 1:** Top 15 prolific countries in Asia

Rank	Country	Total number	Population (million)	Number of publications per million inhabitants	Total citations	Average citations
1	China	1221	1441.66	0.85	18,009	14.92
2	Turkey	562	84.78	6.63	3239	5.76
3	**Israel**	502	9.36	**53.61**	8538	19.95
4	Saudi Arabia	276	35.95	7.68	1891	7.08
5	India	247	1410.00	0.18	3564	14.49
6	Iran	230	87.92	2.62	1549	6.45
7	Japan	200	125.68	1.59	2489	12.2
8	South Korea	190	51.74	3.67	2788	14.01
9	**Singapore**	111	5.45	**20.35**	1270	11.04
10	**Lebanon**	88	5.59	**15.73**	1085	14.66
11	**Kuwait**	72	4.25	**16.94**	938	13.21
12	**Qatar**	42	2.69	**15.62**	448	9.53
13	Malaysia	36	33.57	1.07	333	9.0
14	Jordan	32	11.15	2.87	226	7.29
15	Pakistan	23	231.40	0.10	78	3.71

When adjusted for population, Israel, Singapore, Lebanon, Kuwait, and Qatar emerged as leaders with more than 15 publications per million inhabitants. In contrast, China’s figures were relatively low, with only 0.85 publications per million inhabitants (Table 1). It is notable that China (in Taiwan Province) was the first Asian country to report on BMS (in 1974), and Turkey and Saudi Arabia were early adopters of laparoscopic BMS (in 1995) (Supplementary Table 2).

With respect to annual volume, the top five prolific countries demonstrated consistent growth trends until 2020, the time when COVID-19 emerged, after which a significant decline was noted (as depicted in Fig. 1c).

Within China, the provinces contributing the most were Taiwan, Shanghai, Jiangsu, Beijing, Guangdong, and Shandong, each contributing over 70 articles (Fig. 1d). These provinces are primarily located in the eastern region, where the most productive institutions are situated.

After adjusting for population, Taiwan, Shanghai, Hong Kong, and Beijing demonstrated a relatively higher number of publications per million inhabitants, while Guangdong’s prominence significantly diminished (Supplementary Table 3).

The correlation between research productivity, as gauged by article count, and GDP per capita across countries was found to be weak (*R* = 0.092, *p* = 0.63, Supplementary Fig. 1a). However, within China, a strong correlation was found between research productivity and both regional GDP (*R* = 0.51, *p* < 0.01; Supplementary Fig. 1b), and regional GDP per capita (*R* = 0.74, *p* < 0.001; Supplementary Fig. 1c).

### Top Prolific Institutions

Across the 43-year period, a total of 2914 institutions contributed to BMS research. As shown in Table 2, the majority of the top 20 institutions are universities, with *Tel Aviv University* from Israel leading the group with 181 articles. The second most productive institution was *Min-Sheng General Hospital* from Taiwan, China, contributing 107 articles. Notably, half of these top 20 institutions are affiliated with China.

**Table 2:** Top 20 prolific institutes in Asia

Rank	Institution name	Country	Province (in China)	Number of articles	Citations	Average citations	Universities/hospital
1	Tel Aviv University	Israel		**181**	**3545**	19.59	University
2	Min-Sheng General Hospital	China	Taiwan	107	3231	**30.20**	Hospital
3	Hadassah Hebrew University	Israel		106	2090	19.72	University
4	King Saud University	Saudi Arabia		91	1028	11.30	University
5	Ben-Gurion University of the Negev	Israel		89	1785	20.06	University
6	Iran University of Medical Sciences	Iran		84	349	4.15	University
7	Shanghai Jiao Tong University	China	Shanghai	82	2170	26.46	University
8	Tehran University of Medical Sciences	Iran		74	877	11.85	University
9	The Technion – Israel Institute of Technology	Israel		71	1427	20.10	University
10	I-Shou University	China	Taiwan	69	1141	16.54	University
11	Shahid Beheshti University of Medical Sciences	Iran		69	375	5.43	University
12	National Taiwan University	China	Taiwan	68	2896	42.59	University
13	Central South University	China	Jiangsu	61	638	10.46	University
14	E-Da Hospital	China	Taiwan	59	1083	18.36	Hospital
15	Shandong University	China	Shandong	58	922	15.90	University
16	China Medical University	China	Beijing	57	977	17.14	University
17	University of Health Sciences	Turkey		57	160	2.81	University
18	Jinan University	China	Guangdong	56	499	8.91	University
19	Capital Medical University	China	Beijing	53	267	5.04	University
20	Assuta Medical Center	Israel		52	870	16.73	Hospital

*Tel Aviv University*’s papers were the most frequently cited, amassing 3545 total citations, while *Min-Sheng General Hospital* achieved the highest average citation number per paper (30.20) (Table 2). Supplementary Fig. 2 provides a citation analysis of prolific institutions that have produced at least 20 articles. The figure presents four main clusters: *Cluster 1*, indicated by red nodes, consists mostly of universities from mainland China; *Cluster 2*, denoted by blue nodes, is dominated by institutions from Taiwan, China; C*luster 3*, represented by yellow nodes, contains organizations from Israel; and *Cluster 4*, marked by green nodes, comprises institutes from Iran and Saudi Arabia.

Within China, 744 institutions contributed to BMS research within the study period. The ten most productive of these, mostly universities, are displayed in Supplementary Table 4. *Min-Sheng General Hospital* in Taiwan led with 107 articles, closely followed by *Shanghai Jiao Tong University* in Shanghai with 80 articles. Interestingly, 40% of these top 10 institutions are affiliated with Taiwan, while the remaining are primarily from eastern coastal regions in mainland China.

Supplementary Fig. 3 displays the citation analysis of Chinese institutions that have published at least 20 articles. *Min-Sheng General Hospital*’s papers were the most frequent, with a total of 2975 citations. Collaboration was observed to be stronger within mainland China and Taiwan than inter-regional collaboration.

### Top Prolific Authors

The top 20 prolific authors, based on publication volume, are listed in Supplementary Table 5. The six most productive authors including Lee WJ (143), Lee YC (79), Huang CK (64), Zhang P (62), Chen SC (60), and Wang Y (59) are affiliated with China. Notably, more than half of the top 20 authors are affiliated with China, while the rest are from Japan, Israel, Iran, and India. Lee WJ has the highest citation count (1914) and h-index (41), while Ser KH holds the highest average number of citations per paper (18.19).

**Fig. 2:**
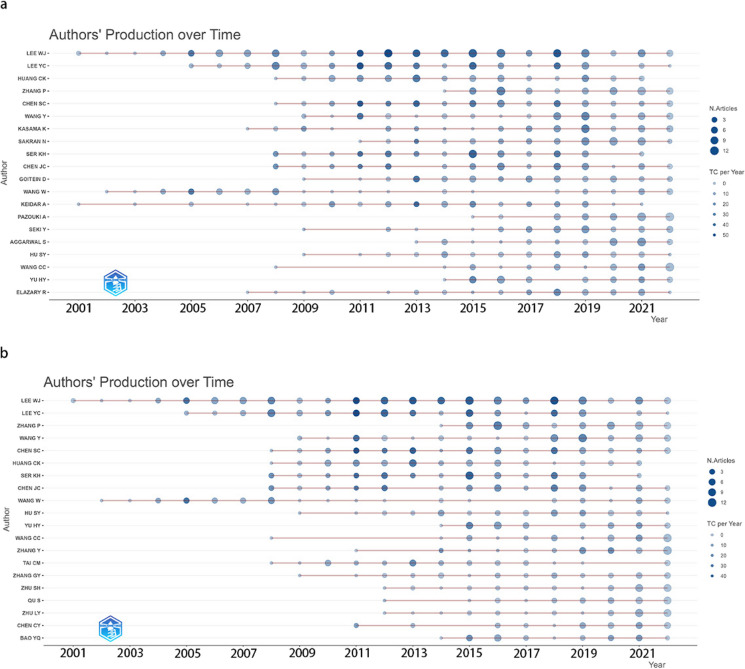
Bubble heat map of author contributions in bariatric metabolic surgery research. **a** Top 20 prolific authors in Asia over time. **b** Top 20 prolific authors in China over time

Figure 2a, a bubble heat map of active authors in Asia, shows an upward trend in the authors’ production volume over time, although annual citations fluctuate. Authors including Lee WJ, Wang W, Keidar A, and Lee YC started contributing before 2005, and have maintained their productivity. Conversely, authors such as Zhang P, Pazouki A, Aggarwal S, and Yu HY entered the field later but have made substantial contributions since. Lee WJ, Lee YC, Ser KH, and Chen SC hold the highest citation levels.

**Fig. 3:**
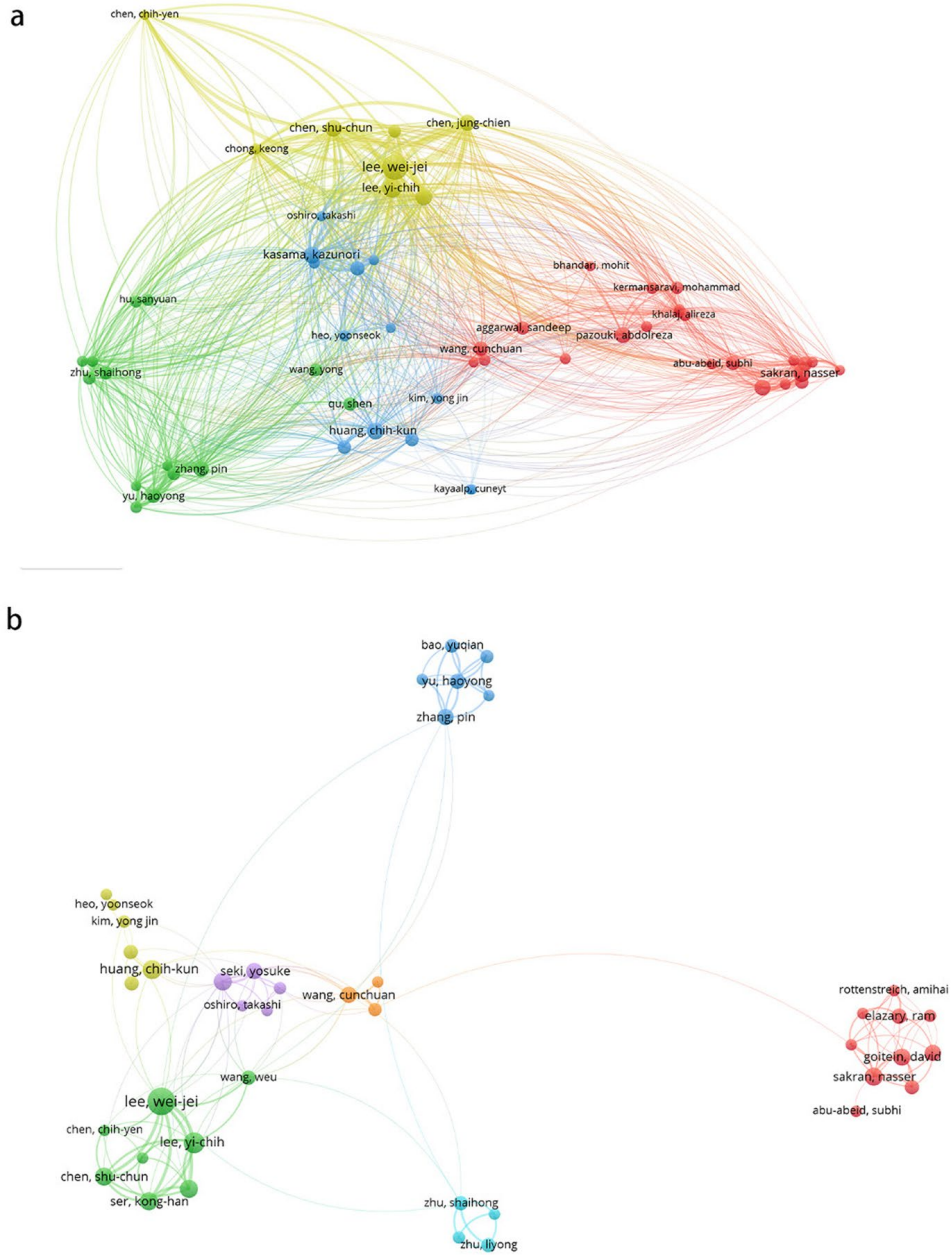
Analysis of active author network in Asia. **a** Citation analysis depicting the impact of active authors in Asia. **b** Co-authorship analysis showcasing the collaborative efforts of active authors in Asia

Figure 3 depicts the active author network map (based on citations and co-authorship) for Asia. It suggests stronger collaboration within regions than between countries, because in general, authors in the same region are represented by nodes of same color, which indicates they have similar research interests and strong collaborations.

**Fig. 4:**
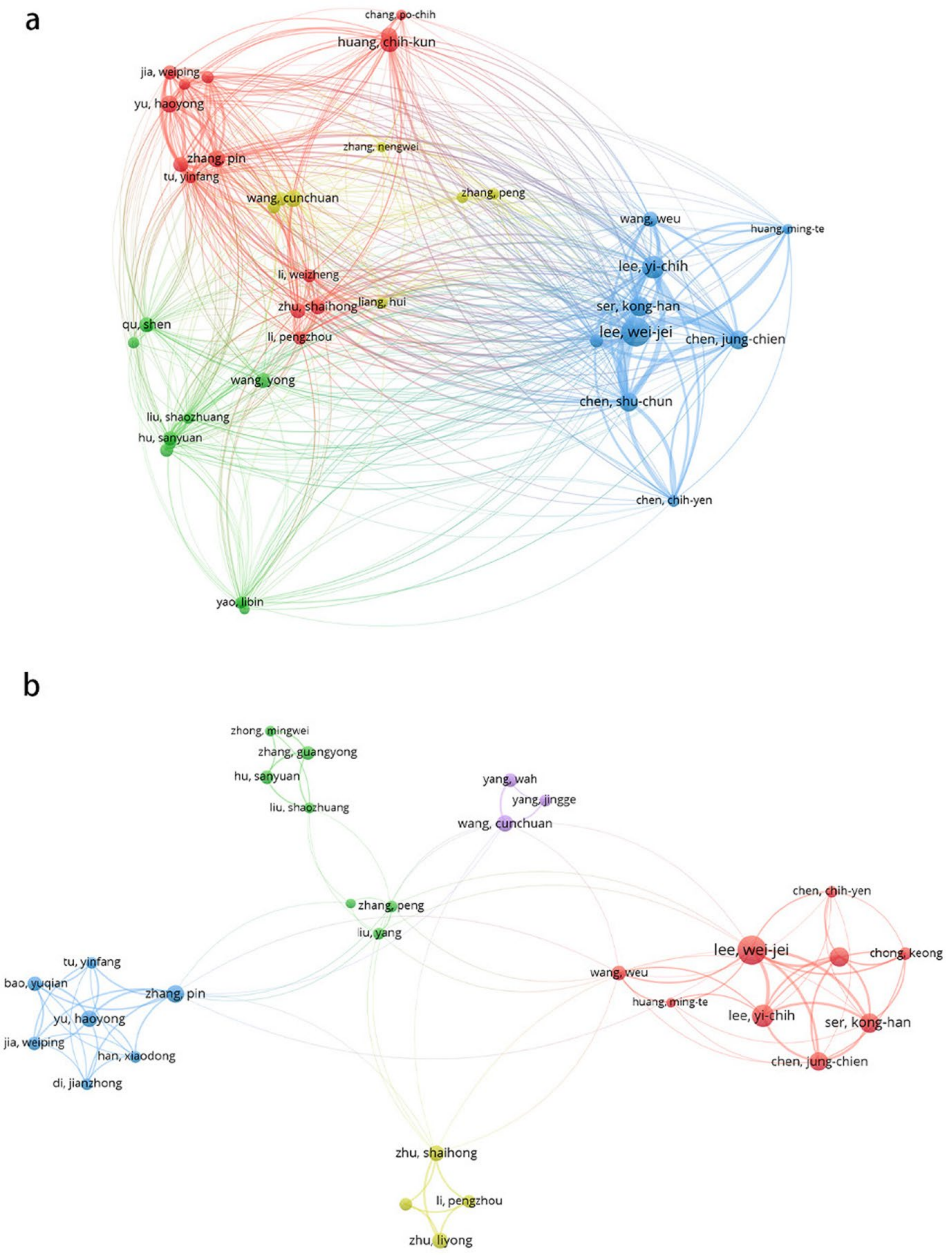
Analysis of active author network in China. **a** Citation analysis reflecting the influence of active authors in China. **b** Co-authorship analysis illustrating the collaborative network of active authors in China

Supplementary Table 6 presents that Lee WJ has received the most citations and h-index in China, while Chen SC has the highest average citation count (15.9). Similar trends as Asia were observed among authors in China (Fig. 2b). The citation and co-authorship analysis (Fig. 4) likewise indicate stronger intra-regional cooperation. The author groups led by Lee WJ and Yu HY are the two most active groups.

In a comparison between Mainland China and Taiwan region (Supplementary Table 7), Taiwan initiated obesity surgery 8 years ahead of Mainland China (in 1974), and also commenced laparoscopic BMS 2 years earlier (in 2000). Despite having fewer publications (*n* = 314) compared with Mainland China (*n* = 856), Taiwan’s per capita publication count, once adjusted for population (13.33), significantly surpasses that of Mainland China (0.61). In terms of top prolific institutions, Mainland China boasts six, while Taiwan has four. Moreover, Taiwan possesses a larger number of prolific authors within Asia.

### Top Prolific Journals

**Fig. 5:**
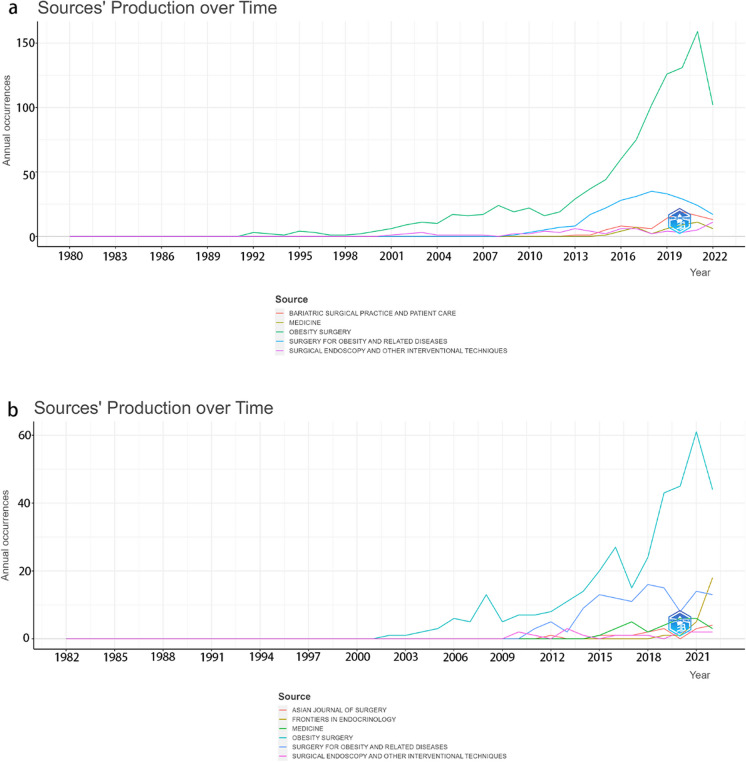
Analysis of active journals in bariatric metabolic surgery research for Asia and China. **a** Yearly article count in the top 5 journals in Asia. **b** Yearly article count in the top 5 journals in China

The 10 most active journals for BMS research in Asia are listed in Supplementary Table 8. *Obesity Surgery* (Q2, 3.479) is the leading journal, with 1078 articles, accounting for roughly 30% of total publications. The second most prolific journal is *Surgery for Obesity and Related Diseases* (Q1, 3.709), making up around 8%. *Obesity Surgery* also boasts the highest h-index (57). As for the annual publication trend for the top five journals, all have seen consistent growth; however, most experienced a decline after 2020 (Fig. 5a).

The 10 most popular journals for BMS research in China are listed in Supplementary Table 9. The two most sought-after journals in China are identical to those in Asia, which are *Obesity Surgery* and *Surgery for Obesity & Related Diseases*, collectively making up 39.72% of total publications. Concurrently, the annual publication trend for the top five journals demonstrates a consistent upward trajectory until 2020 (Fig. 5b).

### Top Cited Articles

Supplementary Table 10 lists the top 20 cited articles, with citation numbers ranging from 42 to 112. The most frequently cited article is “Bariatric Surgery: Asia–Pacific Perspective” written by Lee YJ et al. [8], published in *Obesity Surgery* in 2005, garnering 112 citations. The second most cited article is “Laparoscopic Roux-en-Y Versus Mini-Gastric Bypass for the Treatment of Morbid Obesity: A Prospective Randomized Controlled Clinical Trial” by Lee WJ et al. published in *Annals of Surgery* in 2005, with 111 citations [19]. The third most cited articles were also authored by Lee WJ, published in *Archives of surgery* [20]*.*

In China, the top three popular articles are all works of Lee WJ, with respective local citation counts of 81 [8], 72 [19], 61 [20], and 51 [21]. The most popular one, “Bariatric Surgery: Asia–Pacific Perspective,” provides a comprehensive overview of the development of BMS in Asia–Pacific region [8] (Supplementary Table 11).

### Keyword Analysis

Table 3 lists the most frequently occurred words in title and abstract in Asia and China. The top three words in the title are “*laparoscopic sleeve gastrectomy*,” “*roux-en-y gastric bypass*,” and “*type 2 diabetes mellitus*,” with 607, 337, and 158 occurrences respectively. Meanwhile, the most frequent used three words in the abstract are “*body mass index*” (1323), “*laparoscopic sleeve gastrectomy*” (1217), and “*roux-en-y gastric bypass*” (829). The most frequent three words in title and abstract of China’s publications mirror those in Asia.

**Table 3:** Most frequently occurred words in title and abstract

Asia		China	
**Title’s Words**	**Occurrences**	**Title’s Words**	**Occurrences**
*laparoscopic sleeve gastrectomy*	607	*roux-en-y gastric bypass*	200
*roux-en-y gastric bypass*	337	*laparoscopic sleeve gastrectomy*	143
*type diabetes mellitus*	158	*type diabetes mellitus*	105
morbidly obese patients	124	gastric bypass surgery	67
gastric bypass surgery	105	laparoscopic roux-en-y gastric	56
laparoscopic roux-en-y gastric	98	fatty liver disease	30
adjustable gastric banding	88	body mass index	26
laparoscopic adjustable gastric	80	morbidly obese patients	21
anastomosis gastric bypass	67	laparoscopic bariatric surgery	20
fatty liver disease	65	laparoscopic adjustable gastric	19
**Abstract’s Words**	**Occurrences**	**Abstract’s Words**	**Occurrences**
body mass index	1323	*roux-en-y gastric bypass*	400
laparoscopic sleeve gastrectomy	1217	*body mass index*	391
roux-en-y gastric bypass	829	*laparoscopic sleeve gastrectomy*	254
sleeve gastrectomy lsg	689	type diabetes mellitus	246
mass index bmi	678	mass index bmi	202
morbidly obese patients	546	gastric bypass rygb	195
excess weight loss	524	sleeve gastrectomy lsg	162
type diabetes mellitus	426	diabetes mellitus tdm	160
gastric bypass rygb	353	sleeve gastrectomy sg	147
sleeve gastrectomy sg	331	excess weight loss	132

**Fig. 6:**
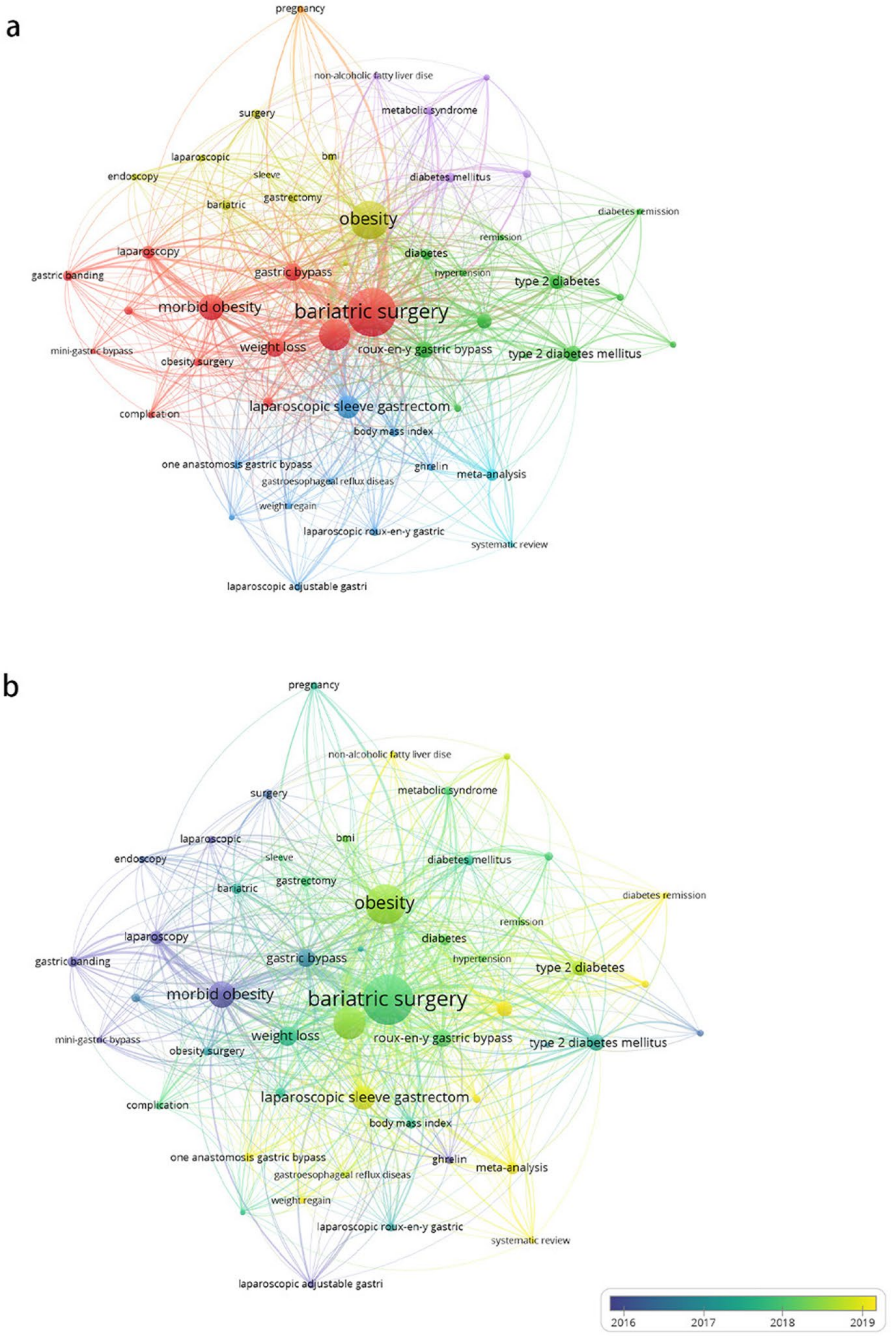
Co-occurrence analysis of author keywords in Asia. **a** Network visualization depicting the relationship between authors keywords in Asia. **b** Overlay visualization showcasing the thematic progression of authors’ keywords in Asia

Figure 6 presents a graphical representation of the co-occurrence of authors’ keywords in Asia. Within this network (Fig. 6a), the most frequently occurring keywords are “*bariatric surgery*,” “*obesity*,” and “*morbid obesity*.”

**Fig. 7:**
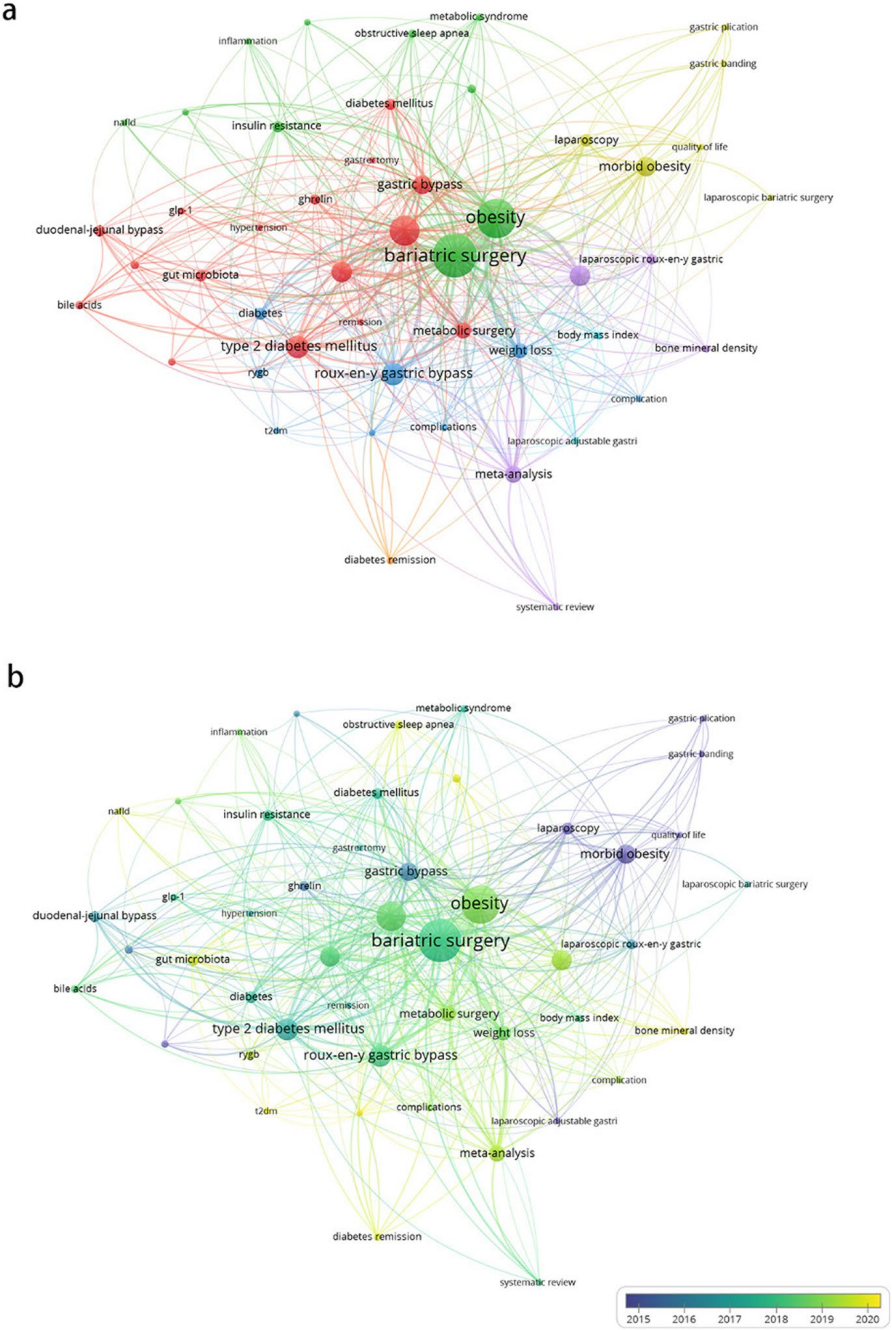
Co-occurrence analysis of author keywords in China. **a** Network visualization illustrating the interconnectivity of authors’ keywords in China. **b** Overlay visualization representing the evolution of research themes based on authors’ keywords in China

Regarding the network overlay (Fig. 6b), the most recent and popular keywords, represented in yellow, include “*laparoscopic sleeve gastrectomy*,” “*meta-analysis*,” and “*one anastomosis gastric bypass*.” The research topic and frontiers in China align with those in Asia (Fig. 7).

**Fig. 8:**
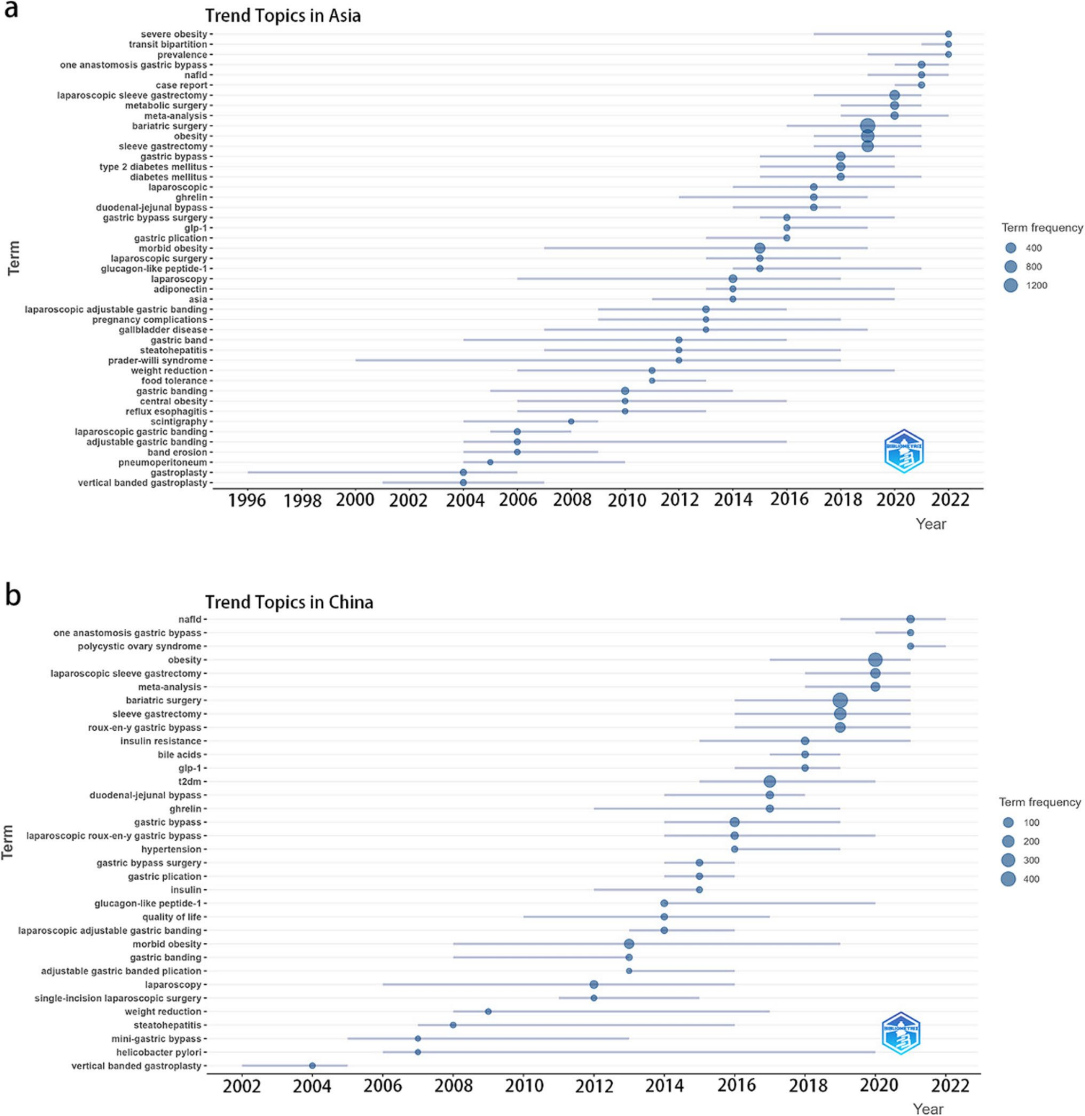
Trend analysis of research topics in bariatric metabolic surgery. **a** Bubble heat map depicting the evolution of research topics in Asia over time. **b** Bubble heat map showcasing the progression of research topics in China over time

Figure 8a displays a graph of trending topics in Asia. ‘‘gastric banding” and “type 2 diabetes” are frequently reported in the initial years. However, in recent years, there has been a shift towards more prevalent topics such as “*laparoscopic sleeve gastrectomy*,” “*one anastomosis gastric bypass*,” and “*nonalcoholic fatty liver disease* or *NAFLD*.”

Similarly, Fig. 8b illustrates the trending topics in China. In recent years, “*nonalcoholic fatty liver disease* or *NAFLD*,” “*one anastomosis gastric bypass*,” “*polycystic ovary syndrome*,” and “*laparoscopic sleeve gastrectomy*” have risen to prominence.

## Discussion

This study offers an in-depth evaluation of the BMS research in Asia, concentrating particularly on China’s substantial contributions during a four-decade period. The study underscores the swift advancement in bariatric research landscape within this region, underlining its notable regional disparities. In particular, the analysis sheds light on the pivotal role populous nations, especially China, have played in driving this scientific development, while acknowledging substantial inputs from diverse institutions, individual authors, and academic journals. Moreover, the study maps the dynamic evolution of trending topics and research frontiers over time, thereby mirroring the continuous transformation of this medical discipline.

Research into BMS has been growing steadily in Asia, with China at the forefront, ever since Taiwan province introduced BMS in the 1970s [8], and Israel published the first related article in 1980 [22]. This surge can be attributed to the rapid economic advancement in the region and an increased public awareness of health issues, fueling the demand for weight loss procedures [11, 23]. Furthermore, the advent of advanced treatment modalities, such as minimally invasive laparoscopic sleeve gastrectomy, could also have played a considerable role [24]. However, the noticeable decline in publication since 2020 can likely be attributed to the reduction in elective surgeries amid the COVID-19 pandemic [25].

The top 10 contributing nations to BMS research are a mix of both developed and developing countries. Affluent nations like Singapore and Japan potentially attribute their significant contributions to high-income levels [23], while the high prevalence of obesity in Middle Eastern countries such as Turkey, Israel, Saudi Arabia, Iran, and Lebanon significantly factors into their research output [26]. In swiftly urbanizing nations like China, escalating obesity rates are seemingly in sync with the pace of urbanization [27], with eastern regions outpacing western ones, reflecting economic disparities. Eastern China, home to several developed provinces or cities such as Taiwan, Shanghai, Guangdong, Beijing, and Jiangsu, along with prestigious universities and hospitals, is better equipped for advanced studies and technological exploration [28]. The correlation between GDP and research productivity seems more prominent in China compared to other Asian countries, potentially due to the relatively higher internal population mobility [29], allowing citizens access to high-quality surgical institutions in developed eastern provinces. This trend underscores the influence of socio-economic factors on the geographical distribution of scientific contributions in the field.

An examination of active authors provides insights into individual contributions and local influence within the field. Early-starting Chinese authors like Lee WJ and Lee YC have published numerous high-impact articles, indicating their productivity and influence. The author’s collaborative network suggests that intra-regional collaborations tend to be stronger than inter-regional ones in Asia and China, possibly owning to cost-saving benefits associated with geographic proximity [30]. Notably, a significant disparity persists in the field of BMS between Mainland China and Taiwan, possibly due to inconsistencies in surgical practices and training methodologies [31]. To mitigate these disparities, fostering robust collaborations among countries, regions, institutions, and researchers is essential. Concurrently, it is crucial for governmental entities and funding bodies to provide ample and consistent financial support, particularly in areas demonstrating low productivity. In the context of China, there is a pressing need to enhance surgeon expertise, improve the quality of clinical data, and facilitate its conversion into scientific research [32]. By adopting these strategies, implementing these measures, we can anticipate significant progress in the domain of BMS within Asia and China, ultimately improving patient outcomes and advancing global obesity management.

Analyzing prolific journals enables the identification of key contributors in the field of BMS. Obesity surgery, the field’s central journal, displays an increasing trend in yearly publication volume. This aligns with previous studies [7], with over 50% of highly cited articles being published in this journal, making it the most influential journal with the highest citations. However, scarcity of BMS research in Q1 journals suggests potential barriers to entry or lack of recognition in the broader medical community. Future research should explore the reasons for this underrepresentation, and researchers should strive to submit their work to high-ranked journals to increase the visibility and impact of BMS research.

The trend topic analysis, coupled with the most frequently occurring terms, reveals crucial trends in BMS research topics. Initially, vertical banded gastroplasty was favored in combating obesity. Minimally invasive surgery via laparoscopy marked a significant turning point [8]. While effective, their impact was often short-lived for a certain patient cohort [33], leading to the increasing popularity of laparoscopic adjustable gastric banding, and subsequently, RYGB [34], offering sustainable weight loss. The advent of laparoscopic sleeve gastrectomy marked a new era, mitigating some complications associated with earlier techniques [35]. When compared to RYGB, it represents a simpler and safer alternative with a decreased likelihood of postoperative complications, including malnutrition and hypoglycemia [36, 37]. The potential advantages of laparoscopic sleeve gastrectomy are numerous and far-reaching within the context of China [38, 39]. Despite the nascent stage of sleeve gastrectomy in China, the procedure has been readily embraced by surgeons, facilitating its incorporation into practice [28, 40]. This preference is driven by lower obesity rates and societal acceptance, with patients favoring safer options [31, 41, 42]. Moreover, the marked improvement in physical condition following the procedure can contribute to reducing prescription drug expenses [43]. Therefore, sleeve gastrectomy has gained favor among both surgeons and patients due to these multiple factors.

In recent years, the exploration of non-alcoholic fatty liver disease (NAFLD) has been an emergent research area globally as well as in Asia. NAFLD, the most prevalent chronic liver disease globally, is particularly prevalent among obese individuals, predominantly in the middle-aged and elderly demographics [44]. Studies suggest that BMS has a positive impact on the pathology of NAFLD and lowers scores of non-invasive assessments such as biochemical parameters and Fibroscan [45–47]. The attention on NAFLD is of vital importance in curbing the health, economic, and social repercussions linked to this condition [48]. As such, it is expected to become a central research focus within the realm of BMS studies, further emphasizing the role of this surgical specialty in managing complex metabolic disorders beyond obesity.

This study is subject to certain limitations. *First and foremost*, relying solely on WoS for data collection may have led to underrepresentation of BMS literature as other bibliographic databases such as Scopus and PubMed were not considered, which could mean that some relevant publications were overlooked. Nevertheless, WoS was selected for its exhaustive coverage and robust citation analysis capabilities. *Second*, only English-language publications were considered, potentially omitting valuable research published in other languages. This language restriction is significant when a substantial portion of research output may arise in non-English speaking countries [49]. *Finally*, bibliometric analysis is inherently retrospective, and while it can provide valuable insights into past and current trends, it may not fully capture and predict future ones. Despite these limitations, this study provides a comprehensive overview of the evolution of BMS research in Asia, with a particular focus on China, contributing to the understanding of the field’s development and trajectory. The findings urge further exploration and enhanced dissemination of BMS research within the region.

## Conclusion

In conclusion, this bibliometric analysis provides a comprehensive insight into the development and contributions of Asia, particularly China, in the realm of BMS research over the past four decades. Our findings highlight a significant growth trajectory within this research field, with discernible contributions from populous countries, institutions, authors, and academic journals. We have also identified the dynamic shifts in key research topics and emerging frontiers over time, mirroring the constantly evolving landscape of BMS. Despite observable regional disparities and a current gap in inter-regional collaborations, the accelerating research output and the improving quality of publications point to a promising future direction. Going forward, enhanced collaborative efforts across different countries and regions, along with better standardization of surgical practices, are recommended to further elevate the quality and global impact of Asia’s, and more specifically, China’s BMS research. This study serves as a valuable reference for future research directions and policy-making within this crucial field. It underscores the importance of this region’s contributions and signals a call to action for fostering a more integrated and collaborative research environment to advance BMS science and practice.

### Supplementary Information

Below is the link to the electronic supplementary material.

Supplementary file1 (PDF 586 KB)

## Data Availability

All data was reported in the manuscript or can be accessed online.

## References

[1] Pi-Sunyer FX (2002). The obesity epidemic: pathophysiology and consequences of obesity. Obes Res.

[2] World Obesity Federation. World Obesity Atlas 2023. https://data.worldobesity.org/publications/?cat=19.

[3] Ramachandran A, Snehalatha C. Rising burden of obesity in Asia. J Obes. 2010;2010:868573. 10.1155/2010/868573. 10.1155/2010/868573 PMC2939400 20871654

[4] Pan X-F, Wang L, Pan A (2021). Epidemiology and determinants of obesity in China. Lancet Diabetes Endocrinol.

[5] Kremen AJ, Linner JH, Nelson CH (1954). An experimental evaluation of the nutritional importance of proximal and distal small intestine. Ann Surg.

[6] Bouldin MJ, Ross LA, Sumrall CD (2006). The effect of obesity surgery on obesity comorbidity. Am J Med Sci.

[7] Dabi Y, Darrigues L, Katsahian S (2016). Publication trends in bariatric surgery: a bibliometric study. Obes Surg.

[8] Lee W-J, Wang W (2005). Bariatric surgery: Asia-pacific perspective. Obes Surg.

[9] Donthu N, Kumar S, Mukherjee D (2021). How to conduct a bibliometric analysis: an overview and guidelines. J Bus Res.

[10] Paolino L, Pravettoni R, Epaud S (2020). Comparison of surgical activity and scientific publications in bariatric surgery: an epidemiological and bibliometric analysis. Obes Surg.

[11] Song Y, Zhao F (2022). Bibliometric analysis of metabolic surgery for type 2 diabetes: current status and future prospects. Updat Surg.

[12] Stefura T, Kacprzyk A, Droś J (2020). The hundred most frequently cited studies on sleeve gastrectomy. Videosurg Miniinvasive Tech.

[13] Toro-Huamanchumo CJ, Morán-Mariños C, Salazar-Alarcon JL (2021). Latin american research on bariatric surgery: a bibliometric study. Obes Surg.

[14] AlRyalat SAS, Malkawi LW, Momani SM. Comparing bibliometric analysis using PubMed, Scopus, and Web of Science databases. J Vis Exp: JoVE. 2019(152). 10.3791/58494. 10.3791/58494 31710021

[15] Sun G, Li L, Zhang X. A visualized and scientometric analysis of research trends of weight loss in overweight/obese children and adolescents (1958–2021). Front Publ Health. 2022:3953. 10.3389/fpubh.2022.928720 PMC9632180 36339176

[16] Aria M, Cuccurullo C (2017). bibliometrix: an R-tool for comprehensive science mapping analysis. J Informet.

[17] Galicia Ernst I, Torbahn G, Schwingshackl L (2022). Outcomes addressed in randomized controlled lifestyle intervention trials in community-dwelling older people with (sarcopenic) obesity—an evidence map. Obes Rev.

[18] Van Eck N, Waltman L (2010). Software survey: VOSviewer, a computer program for bibliometric mapping. Scientometrics.

[19] Lee WJ, Yu PJ, Wang W (2005). Laparoscopic Roux-en-Y versus mini-gastric bypass for the treatment of morbid obesity: a prospective randomized controlled clinical trial. Ann Surg.

[20] Lee WJ, Chong K, Ser KH, et al. Gastric bypass vs sleeve gastrectomy for type 2 diabetes mellitus: a randomized controlled trial. Arch Surg (Chicago, Ill: 1960). 2011;146(2):143–8. 10.1001/archsurg.2010.326. 10.1001/archsurg.2010.326 21339423

[21] Lee WJ, Hur KY, Lakadawala M (2013). Predicting success of metabolic surgery: age, body mass index, C-peptide, and duration score. Surg Obes Relat Dis.

[22] Antal SC, Kovacs ZG (1980). Gastric bypass operation in morbid-obesity. Isr J Med Sci.

[23] Jackson SE, Llewellyn CH, Smith L (2020). The obesity epidemic–nature via nurture: a narrative review of high-income countries. SAGE Open Med.

[24] Zachariah SK, Chang P-C, Ooi ASE (2013). Laparoscopic sleeve gastrectomy for morbid obesity: 5 years experience from an Asian center of excellence. Obes Surg.

[25] Proietti S, Gaboardi F, Giusti G (2020). Endourological stone management in the era of the COVID-19. Eur Urol.

[26] Okati-Aliabad H, Ansari-Moghaddam A, Kargar S, Jabbari N. Prevalence of obesity and overweight among adults in the middle east countries from 2000 to 2020: A systematic review and meta-analysis. J Obes. 2022;2022:8074837. 10.1155/2022/8074837. 10.1155/2022/8074837 PMC8831052 35154826

[27] Du W, Wang H, Su C (2022). Thirty-year urbanization trajectories and obesity in modernizing China. Int J Environ Res Public Health.

[28] Du X, Dai R, Zhou H-X, et al. Bariatric surgery in China: how is this new concept going? Obesity Surg. 2016;26:2906–12. 10.1007/s11695-016-2204-2 27146500

[29] Wang F, Wei X, Liu J (2019). Impact of high-speed rail on population mobility and urbanisation: a case study on Yangtze River Delta urban agglomeration, China. Transp Res A Policy Pract.

[30] Sun Y, Cao C (2015). Intra-and inter-regional research collaboration across organizational boundaries: evolving patterns in China. Technol Forecast Soc Chang.

[31] Yang K, Zhou Y, Wang M (2019). Status of the field of bariatric surgery: a national survey of China in 2018. Obes Surg.

[32] Yang H, Chen Y, Dong Z, et al. Chinese obesity and metabolic surgery database:annual report 2020 (In Chinese). Chin J Obes Metab Dis. 2021;7(01):1–7. http://med.wanfangdata.com.cn/Periodical/zhfpydxdzzz. Accessed 3 May 2023.

[33] Lee WJ, Yu PJ, Wang W, et al. Gastrointestinal quality of life following laparoscopic vertical banded gastroplasty. Obes Surg. 2002;12(6):819–24; discussion 25. https://doi.org/10.1381/096089202320995628 10.1381/096089202320995628 12568188

[34] Wang C, Zhang H, Yu H (2020). Roux-en-Y gastric bypass for T2D treatment in Chinese patients with low BMI: 5-year outcomes. Obes Surg.

[35] Ponce J, DeMaria EJ, Nguyen NT, et al. American society for metabolic and bariatric surgery estimation of bariatric surgery procedures in 2015 and surgeon workforce in the United States. Surg Obes Relat Dis. 2016;12(9):1637–9. 10.1016/j.soard.2016.08.488. 10.1016/j.soard.2016.08.488 27692915

[36] Gu L, Fu R, Chen P (2020). In terms of nutrition, the most suitable method for bariatric surgery: laparoscopic sleeve gastrectomy or Roux-en-Y gastric bypass? A systematic review and meta-analysis. Obes Surg.

[37] Palermo M, Gagner M (2020). Why we think laparoscopic sleeve gastrectomy is a good operation: step-by-step technique. J Laparoendosc Adv Surg Tech.

[38] Halperin F, Ding S-A, Simonson DC (2014). Roux-en-Y gastric bypass surgery or lifestyle with intensive medical management in patients with type 2 diabetes: feasibility and 1-year results of a randomized clinical trial. JAMA Surg.

[39] Rubino F, Nathan DM, Eckel RH (2016). Metabolic surgery in the treatment algorithm for type 2 diabetes: a joint statement by international diabetes organizations. Diabetes Care.

[40] Yang H, Zhang P, Dong Z, et al. (In Chinese). Position statement on recommendations of surgical procedures in metabolic and bariatric surgery of China. Chin J Obes Metab Dis. 2021;7(1):8–12. http://www.sinomed.ac.cn/article.do?ui=202132759. Accessed 3 May 2023.

[41] Wu Y. Overweight and obesity in China. BMJ. 2006;333(7564):362–3. 10.1136/bmj.333.7564.362. 10.1136/bmj.333.7564.362 PMC1550451 16916811

[42] Zeng Q, Li N, Pan X-F (2021). Clinical management and treatment of obesity in China. Lancet Diabetes Endocrinol.

[43] Gould JC, Garren MJ, Starling JR (2004). Laparoscopic gastric bypass results in decreased prescription medication costs within 6 months. J Gastrointest Surg.

[44] Li J, Zou B, Yeo YH (2019). Prevalence, incidence, and outcome of non-alcoholic fatty liver disease in Asia, 1999–2019: a systematic review and meta-analysis. Lancet Gastroenterol Hepatol.

[45] Agarwal L, Aggarwal S, Yadav R (2021). Bariatric surgery in nonalcoholic fatty liver disease (NAFLD): impact assessment using paired liver biopsy and fibroscan. Obes Surg.

[46] Laursen TL, Hagemann CA, Wei C (2019). Bariatric surgery in patients with non-alcoholic fatty liver disease-from pathophysiology to clinical effects. World J Hepatol.

[47] Garg H, Aggarwal S, Yadav R, et al. Utility of transient elastography (fibroscan) and impact of bariatric surgery on nonalcoholic fatty liver disease (NAFLD) in morbidly obese patients. Surg Obes Relat Dis. 2018;14(1):81–91. 10.1016/j.soard.2017.09.005 29126863

[48] Allen AM, Lazarus JV, Younossi ZM. Healthcare and socioeconomic costs of NAFLD: A global framework to navigate the uncertainties. J Hepatol. 2023;79(1):209–17. 10.1016/j.jhep.2023.01.026. 10.1016/j.jhep.2023.01.026 PMC10293095 36740046

[49] Man JP, Weinkauf JG, Tsang M (2004). Why do some countries publish more than others? An international comparison of research funding, English proficiency and publication output in highly ranked general medical journals. Eur J Epidemiol.

