# Supplementary material for: Asia’s Growing Contribution to Obesity Surgery Research: A 40-year Bibliometric Analysis
Source: Obes Surg. 2024 Mar 7;34(6):2139–53. doi: 10.1007/s11695-024-07138-z (PMC11127875; doi:10.1007/s11695-024-07138-z)
Supplement: Supplementary file 1 — Supplementary file1 (PDF 586 KB) [file 11695_2024_7138_MOESM1_ESM.pdf]

Supplementary File

Supplementary Table1. Search Strategy for Web of Science.

#1	TI= ("bariatric surger*" OR "metabolic surger*" OR "obesity surgery" OR "stomach stapling" OR "sleeve gastrectomy" OR "gastric sleeve" OR "gastroplasty" OR "gastric banding" OR "gastric band" OR "Duodenal Switch" OR "biliopancreatic diversion" OR "vertical banded gastroplasty" OR "stomach bypass" OR "gastric bypass" OR "roux-en-y gastric bypass")
#2	AB= ("bariatric surger*" OR "metabolic surger*" OR "obesity surgery" OR "stomach stapling" OR "sleeve gastrectomy" OR "gastric sleeve" OR "gastroplasty" OR "gastric banding" OR "gastric band" OR "Duodenal Switch" OR "biliopancreatic diversion" OR "vertical banded gastroplasty" OR "stomach bypass" OR "gastric bypass" OR "roux-en-y gastric bypass")
#3	AK= ("bariatric surger*" OR "metabolic surger*" OR "obesity surgery" OR "stomach stapling" OR "sleeve gastrectomy" OR "gastric sleeve" OR "gastroplasty" OR "gastric banding" OR "gastric band" OR "Duodenal Switch" OR "biliopancreatic diversion" OR "vertical banded gastroplasty" OR "stomach bypass" OR "gastric bypass" OR "roux-en-y gastric bypass")
#4	(Ti= (animal* OR rat* OR mice)) OR (AB= (animal* OR rat* OR mice)) OR (AK= (animal* OR rat* OR mice))
#5	(#1 OR #2 OR #3) NOT #4

Supplementary Table 2. Distribution of bariatric surgery publications in 29 Asian countries, and the number of publications adjusted for population.

**Countries**	**Number of Articles**	**Populations (million)**	**Number of Publication per million inhabitants**	**Start year of bariatric surgery (laparoscopic)**
China	**1221**	1411.6609	0.85	**1974** (1998)
Mainland China	*856*	*1410*	*0.61*	*1982 (2000)*
Taiwan	*314*	*23.561236*	*13.33*	***1974** (1998)*
Hong Kong	*49*	*7.4131*	*6.61*	*2002 (2002)*
Macao	*2*	*0.686607*	*2.91*	*NA*
Turkey	562	84.775404	6.63	1990 (**1995**)
Israel	502	9.364	53.61	NA
Saudi Arabia	276	35.950396	7.68	1985 (**1995**)
India	247	1410	0.18	2000 (2000)
Iran	230	87.923432	2.62	NA
Japan	200	125.681593	1.59	1982 (2000)
South Korea	190	51.744876	3.67	2003 (2003)
Singapore	111	5.453566	20.35	1987 (2001)
Lebanon	88	5.592631	15.73	
Kuwait	72	4.250114	16.94	1997 (1999)
Qatar	42	2.688235	15.62	2011 (2011)
Malaysia	36	33.573874	1.07	1996 (2001)
Jordan	32	11.148278	2.87	NA
Pakistan	23	231.402117	0.10	NA
Thailand	15	71.601103	0.21	2003 (2005)
Kazakhstan	14	19.000988	0.74	NA
Bahrain	13	1.463265	8.88	NA
Iraq	7	43.533592	0.16	NA
United Arab Emirates	6	9.365145	0.64	2001 (2001)
Philippines	4	113.880328	0.04	2001 (2004)
Bangladesh	2	169.356251	0.01	NA
Palestine	2	NA	NA	NA
Nepal	2	30.034989	0.07	NA
Azerbaijan	2	10.13775	0.20	NA
Indonesia	2	273.753191	0.01	2002 (2002)
Afghanistan	1	40.099462	0.02	NA
Georgia	1	3.70861	0.27	NA
Oman	1	4.520471	0.22	NA

Supplementary Table 3. Regional distribution of bariatric surgery publications in China, and the number of publications adjusted for population.

**Province/Municipal city**	**Total number of publications**	**Number of Population**	**Number of Publications per million inhabitants**
Taiwan	314	23561236	**13.33**
Shanghai	176	24870859	**7.08**
Jiangsu	99	84748016	1.17
Beijing	92	21893059	**4.20**
Guangdong	70	126012510	0.56
Shandong	70	101527453	0.69
Hunan	61	66444864	0.92
Hong Kong	49	7474200	**6.56**
Sichuan	48	83674866	0.57
Liaoning	46	42591407	1.08
Chongqing	36	32054159	1.12
Zhejiang	28	64567688	0.43
Hubei	22	57752557	0.38
Shaanxi	22	39528999	0.56
Jilin	12	24073453	0.50
Anhui	12	61027171	0.20
Fujian	11	41540086	0.26
Heilongjiang	8	31850088	0.25
Tianjin	7	13866009	0.50
Gansu	7	25019831	0.28
Henan	6	99365519	0.06
Shanxi	5	34915616	0.14
Jiangxi	5	45188635	0.11
Xinjiang	3	25852345	0.12
Yunnan	3	47209277	0.06
Hebei	2	74610235	0.03
Guangxi	2	50126804	0.04
Macao	2	683218	2.93
Hainan	1	10081232	0.10
Guizhou	1	38562148	0.03
Inner Mongolia	1	24049155	0.04
Qinghai	0	5923957	0.00
Ningxia	0	7202654	0.00
Xizang	0	3648100	0.00

Supplementary Table 4. Top 10 prolific institutions in China.

Rank	Institution name	Province/ Municipal City	Number of Articles	Citations	Average Citations	Universities/Hospital
1	Min-Sheng General Hospital	Taiwan	107	2975	27.80	Hospital
2	Shanghai Jiao Tong University	Shanghai	80	2157	26.96	University
3	I-Shou University	Taiwan	69	1144	16.58	University
4	National Taiwan University	Taiwan	67	2864	42.75	University
5	Central South University	Hunan	61	646	10.59	University
6	E-Da Hospital	Taiwan	57	995	17.46	Hospital
7	Shandong University	Shandong	57	875	15.35	University
8	China Medical University	Liaoning	56	974	17.39	University
9	Jinan University	Guangdong	53	460	8.68	University
10	Capital Medical University	Beijing	52	264	5.08	University

Supplementary Table 5. Top 20 prolific authors in Asia.

Authors	Affiliation	Countries	Articles	Citations	Average citations	h-index
LEE WJ	Min-Sheng General Hospital	China	**143**	**1914**	13.38	**41**
LEE YC	Chien Hsin University of Science and Technology	China	79	1206	15.27	33
HUANG CK	E-Da Hospital	China	64	422	6.59	24
ZHANG P	Shanghai Jiao Tong University	China	62	231	3.73	13
CHEN SC	Kaohsiung Medical University Hospital	China	60	954	15.90	28
WANG Y	The Second Hospital of Anhui Medical University	China	59	131	2.22	15
KASAMA K	Yotsuya Medical Cube	Japan	56	617	11.02	20
SAKRAN N	Bar-Ilan University	Israel	56	218	3.89	17
SER KH	Min-Sheng General Hospital	China	54	982	**18.19**	29
CHEN JC	Taichung Tzu Chi Hospital	China	53	755	14.25	23
GOITEIN D	Tel Aviv	Israel	50	251	5.02	19
PAZOUKI A	Iran University of Medical Sciences	Iran	50	48	0.96	8
WANG W	En-Chu-Kong Hospital	China	48	600	12.50	20
KEIDAR A	Assuta Ashdod Public Hospital	Israel	47	314	6.68	26
SEKI Y	Yotsuya Medical Cube	Japan	43	385	8.95	15
AGGARWAL S	Pandit Bhagwat Dayal Sharma Postgraduate Institute of Medical Sciences	India	40	86	2.15	11
HU SY	Shandong University	China	40	164	4.10	13
WANG CC	The First Affiliated Hospital of Jinan University	China	40	112	2.80	10
YU HY	Sun Yat-sen University	China	40	177	4.43	13
ELAZARY R	Hadassah-Hebrew University Medical Center	Israel	39	197	5.05	17

Supplementary Table 6. Top 20 prolific authors in China.

Authors	Province	Articles	Citations	Average citations	h-index
LEE WJ	Taiwan	**143**	**1941**	13.6	**41**
LEE YC	Taiwan	79	1206	15.3	33
HUANG CK	Taiwan	64	422	6.6	24
ZHANG P	Guangdong	62	231	3.7	13
CHEN SC	Taiwan	60	954	**15.9**	28
WANG Y	Anhui	59	131	2.2	15
SER KH	Taiwan	54	982	18.2	29
CHEN JC	Taiwan	53	755	14.2	23
WANG W	Taiwan	48	600	12.5	20
WANG CC	Guangdong	40	112	2.8	10
YU HY	Guangdong	40	177	4.4	13
HU SY	Shandong	40	164	4.1	13
ZHANG Y	Shanghai	37	138	3.7	11
TAI CM	Taiwan	35	260	7.4	20
ZHANG GY	Shandong	35	112	3.2	11
ZHU SH	Hunan	35	78	2.2	8
QU S	Shanghai	34	62	1.8	9
ZHU LY	Hunan	34	75	2.2	8
CHEN CY	Taiwan	32	182	5.7	10
BAO YQ	Shanghai	31	122	3.9	12

Supplementary Table 7. Comparative analysis between mainland China and Taiwan Province.

	**Mainland China**	**Taiwan**
Start year of bariatric surgery (laparoscopic)	1982 (2000)	1974 (1998)
Number of Publications	856	314
Number of Publications per capita	0.61	13.33
Number of top prolific institutions in Asia	6	4
% of top prolific institutions in China	60%	40%
Number of top prolific authors in Asia	5	7
% of top prolific authors in China	55%	45%

Supplementary Table 8. Top 10 prolific journals in Asia.

**Journals**	**Number of Articles**	**Citations**	**Average Citations**	**Quartile Scores**	**IF/2021**	**h-index**
Obesity Surgery	1078	17876	16.58	Q2	3.479	57
Surgery for Obesity & Related Diseases	260	4198	16.15	Q1	3.709	32
Bariatric Surgical Practice and Patient Care	96	108	1.13	Q4	0.368	4
Surgical Endoscopy and Other Interventional Techniques	71	1836	25.86	Q2	3.453	25
Medicine	46	412	8.96	Q3	1.817	10
Surgical Laparoscopy Endoscopy & Percutaneous Techniques	40	366	9.15	Q4	1.455	10
International Journal of Surgery Case Reports	39	44	1.13	Q4	0.63	3
JOURNAL OF MINIMAL ACCESS SURGERY	37	261	7.05	Q4	1.018	11
Frontiers In Endocrinology	35	216	6.17	Q1	6.055	7
Journal of Laparoendoscopic & Advanced Surgical Techniques	34	251	7.38	Q3	1.766	10

Supplementary Table 9. Top 10 prolific journals in China.

Rank	Journals	Number of Articles	Citations	Average Citations	Quartile Scores	Impact factor	h-index
1	Obesity Surgery	364	**6286**	**17.27**	Q2	3.479	**41**
2	Surgery for Obesity & Related Diseases	121	1846	15.26	Q1	3.709	22
3	Medicine	30	300	10.00	Q3	1.817	8
4	Frontiers In Endocrinology	25	45	1.80	Q1	6.055	4
5	Surgical Endoscopy and Other Interventional Techniques	16	367	22.94	Q2	3.453	9
6	Diabetes Metabolic Syndrome and Obesity-Targets and Therapy	15	24	1.60	Q3	3.249	3
7	Asian Journal of Surgery	16	105	6.56	Q2	2.808	5
8	Scientific Reports	13	69	5.31	Q2	4.997	5
9	BMC Surgery	12	130	10.83	Q3	2.03	4
10	HEPATO-GASTROENTEROLOGY	12	47	3.92	Q4	0.792	7

Supplementary Table 10. Top 20 cited articles in Asia.

Title	First Author	Journal	Country	Affiliation	IF/2021	Year	Local citations
Bariatric Surgery: Asia-Pacific Perspective	LEE WJ	OBES SURG	China	Min-Sheng General Hospital	3.479	2005	112
Laparoscopic Roux-en-Y Versus Mini-Gastric Bypass for the Treatment of Morbid Obesity: A Prospective Randomized Controlled Clinical Trial	LEE WJ	ANN SURG	China	Min-Sheng General Hospital	13.787	2005	111
Gastric Bypass vs Sleeve Gastrectomy for Type 2 Diabetes Mellitus	LEE WJ	ARCH SURG-CHICAGO	China	Min-Sheng General Hospital	4.926	2011	96
Predicting success of metabolic surgery: age, body mass index, C-peptide, and duration score	LEE WJ	SURG OBES RELAT DIS	China	Min-Sheng General Hospital	3.709	2013	87
IFSO-APC Consensus Statements 2011	KASAMA K	OBES SURG	Japan	Yotsuya Medical Cube	3.479	2012	68
Laparoscopic Roux-en-Y Vs. Mini-gastric Bypass for the Treatment of Morbid Obesity: a 10-Year Experience	LEE WJ	OBES SURG	China	Min-Sheng General Hospital	3.479	2012	66
Effect of Laparoscopic Mini-Gastric Bypass for Type 2 Diabetes Mellitus: Comparison of BMI >35 and <35 kg/m^2	LEE WJ	J GASTROINTEST SURG	China	Min-Sheng General Hospital	3.267	2008	58
One Thousand Consecutive Mini-Gastric Bypass: Short- and Long-term Outcome	NOUN R	OBES SURG	Lebanon	Hôtel-Dieu de France Hospital and University Saint Joseph Medical School	3.479	2012	58
Laparoscopic Sleeve Gastrectomy with Duodenojejunal Bypass: Technique and Preliminary Results	KASAMA K	OBES SURG	Japan	Yotsuya Medical Cube	3.479	2009	57
Results of laparoscopic sleeve gastrectomy (LSG) at 1 year in morbidly obese Korean patients	HAN SM	OBES SURG	South Korea	Kangnam CHA Hospital	3.479	2005	55
Laparoscopic Sleeve Gastrectomy—Volume and	YEHOSHUA	OBES SURG	Israel	Tel Aviv University	3.479	2008	55

Pressure Assessment	RT						
Short-term Results of Laparoscopic Mini-Gastric Bypass	WANG W	OBES SURG	China	En-Chu-Kong Hospital	3.479	2005	48
Gastric Bypass, and Mini-Gastric Bypass, to Determine an Effective and Safe Bariatric and Metabolic Procedure	JAMMU GS	OBES SURG	India	Jammu Hospital	3.479	2016	48
Laparoscopic Roux-en-Y Gastric Bypass for the Treatment of Type II Diabetes Mellitus in Chinese Patients with Body Mass Index of 25–35	HUANG CK	OBES SURG	China	E-Da Hospital,	3.479	2011	47
Laparoscopic sleeve gastrectomy for type 2 diabetes mellitus: predicting the success by ABCD score	LEE WJ	SURG OBES RELAT DIS	China	Min-Sheng General Hospital	3.709	2015	47
Predicting the Glycemic Response to Gastric Bypass Surgery in Patients With Type 2 Diabetes	DIXON JB	DIABETES CARE	Australia	Baker IDI Heart and Diabetes Institute	17.155	2013	46
Bariatric Surgery in Asia in the Last 5 Years (2005–2009)	LOMANTO D	OBES SURG	Singapore	Minimally Invasive Surgical Centre	3.479	2011	43
Gastric leaks after sleeve gastrectomy: a multicenter experience with 2,834 patients	SAKRAN N	SURG ENDOSC	Israel	Bar-Ilan University	3.453	2013	43
Laparoscopic sleeve gastrectomy for diabetes treatment in nonmorbidly obese patients: Efficacy and change of insulin secretion	LEE WJ	SURGERY	China	Min-Sheng General Hospital	4.348	2010	42
Changes in postprandial gut hormones after metabolic surgery: a comparison of gastric bypass and sleeve gastrectomy	LEE WJ	SURG OBES RELAT DIS	China	Min-Sheng General Hospital	3.709	2011	42

Supplementary Table 11. Top 20 cited articles in China.

Title	First Author	Journal	Country/ Region	Affiliation	IF/2021	Year	Local citations
Bariatric Surgery: Asia-Pacific Perspective	LEE WJ	OBES SURG	Taiwan	Min-Sheng General Hospital	3.479	2005	81
Laparoscopic Roux-en-Y Versus Mini-Gastric Bypass for the Treatment of Morbid Obesity: A Prospective Randomized Controlled Clinical Trial	LEE WJ	ANN SURG	Taiwan	Min-Sheng General Hospital	13.787	2005	72
Gastric Bypass vs Sleeve Gastrectomy for Type 2 Diabetes Mellitus A Randomized Controlled Trial	LEE WJ	ARCH SURG-CHICAGO	Taiwan	Min-Sheng General Hospital	4.926	2011	61
Predicting success of metabolic surgery: age, body mass index, C-peptide, and duration score	LEE WJ	SURG OBES RELAT DIS	Taiwan	Min-Sheng General Hospital	3.709	2013	51
Laparoscopic Roux-en-Y Gastric Bypass for the Treatment of Type II Diabetes Mellitus in Chinese Patients with Body Mass Index of 25–-35	HUANG CK	OBES SURG	Taiwan	E-Da Hospital	3.479	2011	40
Effect of Laparoscopic Mini-Gastric Bypass for Type 2 Diabetes Mellitus: Comparison of BMI >35 and <35 kg/m^2	LEE WJ	J GASTROINTEST SURG	Taiwan	Min-Sheng General Hospital	3.267	2008	39
Changes in postprandial gut hormones after metabolic surgery: a comparison of gastric bypass and sleeve gastrectomy	LEE WJ	SURG OBES RELAT DIS	Taiwan	Min-Sheng General Hospital	3.709	2011	35

Experience in laparoscopic sleeve gastrectomy for morbidly obese Taiwanese: staple-line reinforcement is important for preventing leakage	SER KH	SURG ENDOSC	Taiwan	Min-Sheng General Hospital	3.453	2010	33
Laparoscopic sleeve gastrectomy for diabetes treatment in nonmorbidly obese patients: Efficacy and change of insulin secretion	LEE WJ	SURGERY	Taiwan	Min-Sheng General Hospital	4.348	2010	32
Short-term Results of Laparoscopic Mini-Gastric Bypass	WANG W	OBES SURG	Taiwan	En-Chu-Kong Hospital	3.479	2005	29
Visceral fat area as a new predictor of short-term diabetes remission after Roux-en-Y gastric bypass surgery in Chinese patients with a body mass index less than 35 kg/m^2	YU HY	SURG OBES RELAT DIS	Taiwan	Min-Sheng General Hospital	3.709	2015	29
Predicting the Glycemic Response to Gastric Bypass Surgery in Patients With Type 2 Diabetes	DIXON JB	DIABETES CARE	Australia	Baker IDI Heart and Diabetes Institute	17.155	2013	26
Laparoscopic sleeve gastrectomy for type 2 diabetes mellitus: predicting the success by ABCD score	LEE WJ	SURG OBES RELAT DIS	Taiwan	Min-Sheng General Hospital	3.709	2015	25
Effects of Obesity Surgery on the Metabolic Syndrome	LEE WJ	ARCH SURG-CHICAGO	Taiwan	Min-Sheng General Hospital	4.926	2004	24

Effect of laparoscopic Roux-en-Y gastric bypass surgery on type 2 diabetes mellitus with hypertension: A randomized controlled trial	LIANG ZW	DIABETES RES CLIN PR	Japan	Yotsuya Medical Cube	8.18	2013	24
Gastrointestinal Quality of Life Following Laparoscopic Adjustable Gastric Banding in Asia	LEE WJ	OBES SURG	Taiwan	Min-Sheng General Hospital	3.479	2006	23
Laparoscopic Roux-en-Y Vs. Mini-gastric Bypass for the Treatment of Morbid Obesity: a 10-Year Experience	LEE WJ	OBES SURG	Taiwan	Min-Sheng General Hospital	3.479	2012	22
Laparoscopic Mini-gastric Bypass: Experience with Tailored Bypass Limb According to Body Weight	LEE WJ	OBES SURG	Taiwan	Min-Sheng General Hospital	3.479	2008	21
Diabetes Remission and Insulin Secretion After Gastric Bypass in Patients with Body Mass Index <35 kg/m^2	LEE WJ	OBES SURG	Taiwan	Min-Sheng General Hospital	3.479	2011	21
C-peptide Predicts the Remission of Type 2 Diabetes After Bariatric Surgery	LEE WJ	OBES SURG	Taiwan	Min-Sheng General Hospital	3.479	2012	21

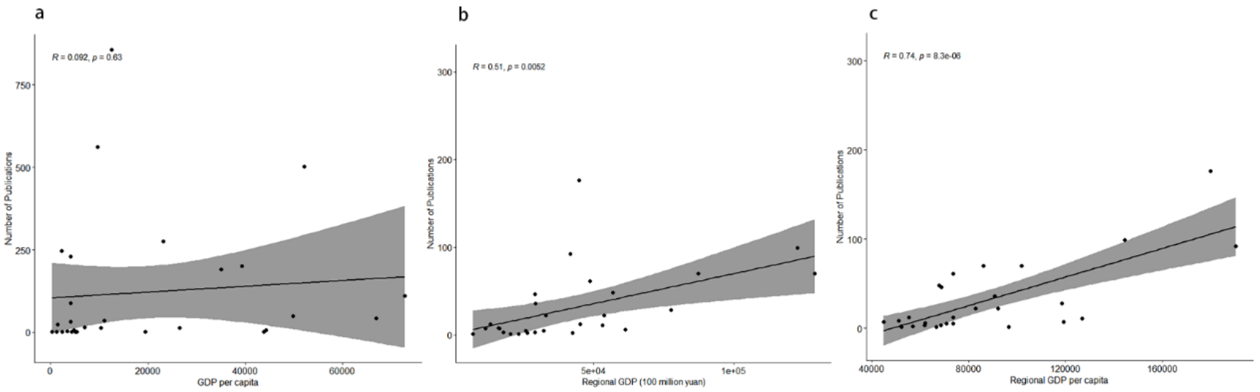

Supplementary Figure 1. Correlation analysis between economic indicators and research productivity. a) Correlation between GDP and research productivity across Asian countries. b) Correlation between regional GDP and research productivity across Chinese provinces and municipal cities. c) Correlation between GRP per capita and research productivity across Chinese provinces and municipal cities.

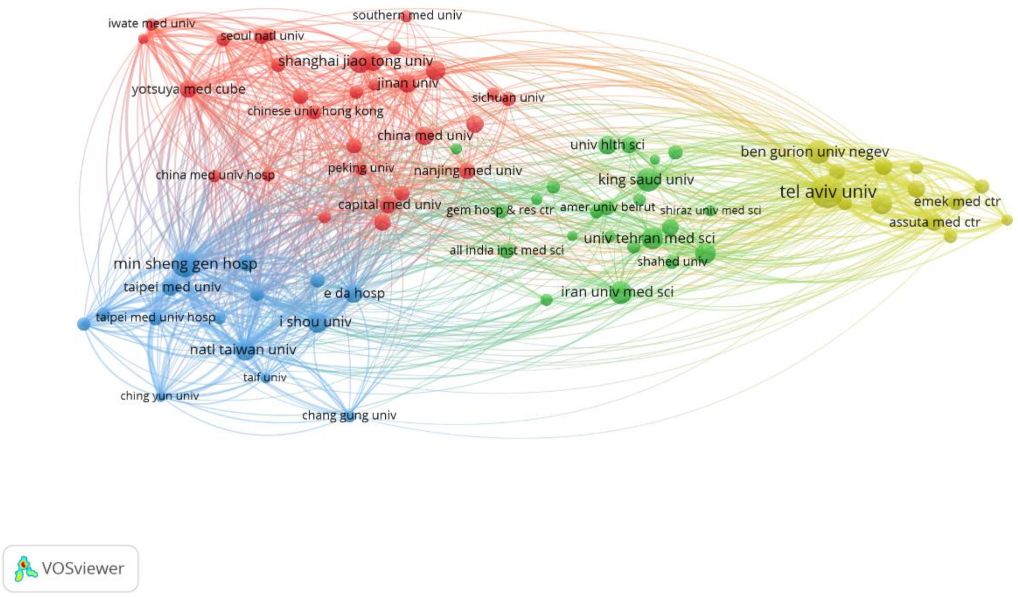

Supplementary Figure 2. Citation analysis of active institutions in Asia.

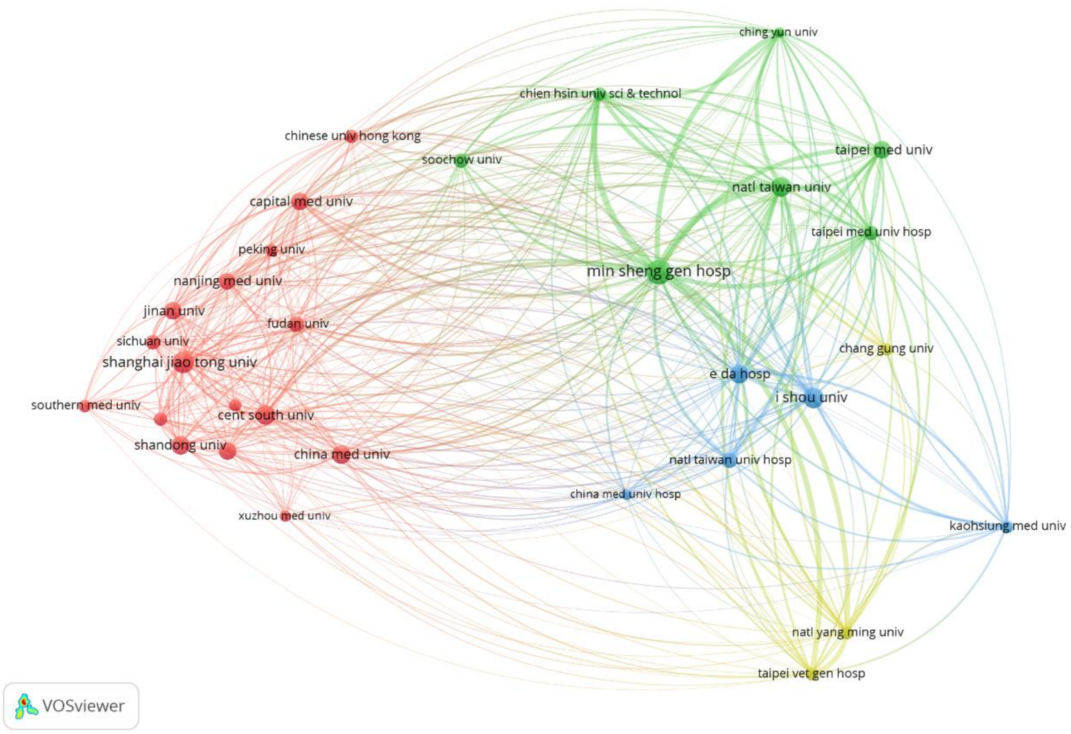

Supplementary Figure 3. Citation analysis of active institutions in China.
